# Coronavirus disease 2019 (COVID-19) and pediatric gastroenterology 

**Published:** 2020

**Authors:** Pejman Rohani, , Sara Ahmadi Badi, Arfa Moshiri, Seyed Davar Siadat

**Affiliations:** 1 *Pediatric Gastroenterology, Hepatology and Nutrition Research Center, Research Institute for Children Health, Shahid Beheshti Medical University, Tehran, Iran*; 2 *Microbiology Research Center, Pasteur Institute of Iran, Tehran, Iran*; 3 *Mycobacteriology and Pulmonary Research Department, Pasteur Institute of Iran, Tehran, Iran*; 4 *Gastroenterology and Liver Diseases Research Center, Research Institute for Gastroenterology and Liver Diseases, Shahid Beheshti University of Medical Sciences, Tehran, Iran *

**Keywords:** SARS-CoV-2, Pediatric COVID-19, Gastroenterology

## Abstract

The coronavirus disease 2019 (COVID-19) is responsible for the new pandemic, which remains an important health and economic challenge worldwide. The causative agent is a novel coronavirus called severe acute respiratory syndrome coronavirus 2 (SARS-CoV-2), which is similar to SARS-CoV-1 and Middle East respiratory syndrome coronavirus (MERS-CoV). Adult infection with respiratory symptoms was considered in the beginning of the pandemic. Now, it has been reported that SARS-CoV-2 infects children and other organs such as the gastrointestinal tract. SARS-CoV-2 enters the host cells through angiotensin converting enzyme-2 (ACE2) receptors as the main receptor expressed in various organs such as the lungs and gastrointestinal tract. Studies on children and the clinical manifestations of COVID-19 do not completely explain the natural course of infection in children, and precisely how the GI tract is involved is not understood. The present article highlights the gastrointestinal manifestations and pathological findings in children with COVID-19. According to the evidence, SARS-CoV-2 infection is milder in children and may present different clinical symptoms from adults. Common clinical manifestations of pediatric COVID-19 include cough, fever, sore throat, malaise, fatigue, and GI symptoms such as diarrhea, abdominal pain, nausea, and vomiting. Furthermore, liver and pancreatic enzymes may be elevated during the pediatric COVID-19 course. Asymptomatic children carriers are potential sources of infection for adults, especially elderly ones. Diagnosis, treatment, and isolation of children are the most effective ways to control the expansion of the COVID-19 pandemic.

## Introduction

 The coronavirus disease 2019 (COVID-19) pandemic, caused by severe acute respiratory syndrome coronavirus 2 (SARS-CoV-2), remains an important health and economic concern worldwide (1). The first report of COVID-19 was likely contracted from a zoonotic source in China in December 2019, and then the disease spread rapidly to other countries with 27,032,617 confirmed cases of COVID-19 on September, 2020, according to the World Health Organization (WHO). The first focus of COVID-19 was adults who demonstrated symptoms of respiratory tract infection as with other respiratory coronaviruses, i.e. SARS-CoV-1 and Middle East respiratory syndrome coronavirus (MERS-CoV) (2). Later, infection in children and the involvement of other organs such as the gastrointestinal (GI) tract were noted by physicians. Studies on children and the clinical manifestations of COVID-19 do not explain the natural course of infection in children, and precisely how the GI tract is involved remains unknown. According to data from China and the United States, infection is milder in children and may have different clinical presentations than in adults (3, 4).

**Mechanism of gastrointestinal disease**

Similar to MERS-CoV and SARS-CoV-1, SARS-CoV-2 belongs to the β-coronaviruses which are positive single-stranded RNA viruses enclosed in envelopes containing glycosylated spike (S) proteins (5). The entry of SARS-CoV-2 to the host cells is mediated by the binding of S proteins to angiotensin converting enzyme-2 (ACE2) receptors. In addition, the fusion of the virus to cells needs transmembrane protease serine 2 (TMPRSS2), which helps the detachment of the S protein of the virus from the cell membrane (6). A structural study reported the higher affinity of SARS-CoV-2 to ACE2 compared to SARS-CoV, which could explain the higher COVID-19 infection rate (7). There are many ACE2 receptors in the GI tract, from the esophagus through the colon. The pancreas and cholangiocytes also have ACE2 receptors (8). According to the data, the GI tract, liver, and pancreas can be suitable targets for SARS-CoV-2. The presence of SARS-CoV-2 in intestinal mucosa is evidence of viral invasion. Despite virus shedding in the stools of patients with COVID-19, fecal-oral transmission is a suspected yet currently unproven route of transmission (9, 10). 

Many studies have reported the clinical presentations of COVID-19 infection, and some have mentioned GI manifestations (Table 1). Diarrhea, nausea, vomiting, and abdominal pain are common GI manifestations with diarrhea being the most common. Liguoro et al. reported the incidence of diarrhea and vomiting to be 9.7% and 7.2%, respectively (11). Anorexia or loss of appetite could be a result of systemic inflammation which generally occurs as a common symptom of most infections; thus, in our opinion, anorexia is not a GI manifestation of COVID-19 infection. GI manifestations could present before other symptoms, and in some studies, they have been reported as the only manifestations. A study on adults indicated that GI manifestations may be a sign of worse prognoses of COVID-19 infection (12). 

During the COVID-19 pandemic, one important concern is pediatric patients with inflammatory bowel disease (IBD) due to the mechanism of GI involvement in COVID-19 patients. Although it is known that there are a lot of ACE2 receptors in the ileum and colon, how IBD affects ACE2 expression has not been determined. Because many COVID-19 patients take immunosuppressive medication, such as steroids, immunomodulators, or biologic agents, an important question has arisen about the tendency of patients to COVID-19 infection or even more severe ones. Turner et al. have declared that currently the evidence is not sufficient to show that pediatric patients are more prone to infection or a worse course of disease (13). Current studies suggest that no change in medication or reduction in immunosuppression is mandatory. Any change in the treatment protocol may predispose patients to a relapse, creating a need for more immunosuppression and predisposing patients to infection or hospital admission (13, 14). 

**Hepatobiliary and pancreatic manifestations**

Several reports have demonstrated elevated liver enzymes in children infected with SARS-Cov-2 (Table 2). Liguoro et al. mentioned high transaminase in 12.3% children infected with COVID-19 (11). Studies on adults have indicated aspartate aminotransferase elevation concurrent with severe disease (15). Acute hepatitis and direct hyperbilirubinemia are not unexpected while ACE2 receptors present themselves on cholangiocytes (16). 

**Table 1:** GI manifestations in children infected with SARS-CoV-2

Study Population	Nausea/Vomiting Patient/Percentage	Abdominal pain Patient/Percentage	Diarrhea Patient/Percentage	Reference
291	31 (11)	17 (5.8)	37 (13)	(21)
20	2 (10)	0	3 (15)	(22)
171	11 (6.4)	0	15 (8.8)	(23)
6	4 (47)	0	0	(24)
8	4 (50)	0	3 (38)	(25)
25	2 (8)	0	3 (12)	(26)
10	0	0	3 (30)	(10)
31	0	0	3 (10)	(27)
36	2 (5)	0	2 (5)	(28)
34	4 (12)	0	4 (12)	(29)
9	0	0	2 (22)	(30)
7	0	0	4 (57)	(31)
100	10 (10)	0	9 (9)	(32)

**Table 2:** Elevated liver enzymes in children infected with SARS-Cov-2

Study Population	Patient/Percentage	Reference
171	25 (15)	(23)
20	5 (25)	(22)
8	2 (25)	(25)
10	2 (20)	(33)
31	6 (19)	(27)
2	2 (100)	(34)
36	3 (8)	(28)
100	10 (10)	(32)
10	2 (20)	(35)

Acute pancreatitis has not been reported in children. Wang et al. mentioned that among 52 adult patients with COVID-19 pneumonia, 17% had elevated levels of pancreatic enzymes (17). To diagnose acute pancreatitis, radiologic findings (abdominal CT or abdominal ultrasound) are necessary; elevated pancreatic enzymes may not imply pancreatic injury. According to Baltar et al., hyperlipasemia is reported in a minority of adult patients with COVID-19 infection, and it has not been associated with worse prognoses. In their study, the radiologic findings of patients were not consistent with a diagnosis of pancreatitis (18). 

**Endoscopic procedures during COVID-19**

In their position paper about pediatric endoscopy during the COVID-19 pandemic, The North American Society of Pediatric Gastroenterology, Hepatology, and Nutrition (NASPGHAN) declared that upper and lower endoscopy are very high-risk procedures for healthcare workers (19) and should be conducted only in limited urgent situations. During the procedures, proper precautions for healthcare workers must be taken, including the use of face respirators such as the FFP2 or N95 mask, face shields, hair nets, water-proof disposable gowns, double latex gloves, and shoe covers. In addition, the procedure room should have a negative pressure system (20). SARS-CoV-2 infection is common in children who may have different clinical presentations from adults. Although children are usually asymptomatic carriers and have a good prognosis, it is noteworthy that they are potential sources of infection, especially for older adults. Diagnosis, treatment, and isolation of infected children are the most effective ways to control the spread of the COVID-19 pandemic. A COVID-19 diagnosis should be considered in children with common clinical manifestations, including cough, fever, sore throat, malaise, fatigue, and GI symptoms such as diarrhea, abdominal pain, nausea, and vomiting. The course of pediatric infection is usually milder than adults, but severe cases may occur. Elevated liver and pancreatic enzymes may also be detected during the course of pediatric COVID-19. Supportive care is usually the only treatment needed. However, this interpretation needs more epidemiological and clinical investigation because of the rapidly growing amount of released data 

## Conflict of interests

The authors declare that they have no conflict of interest.
